# OUTdoor Swimming as a nature-based Intervention for DEpression (OUTSIDE): study protocol for a feasibility randomised control trial comparing an outdoor swimming intervention to usual care for adults experiencing mild to moderate symptoms of depression

**DOI:** 10.1186/s40814-023-01358-3

**Published:** 2023-07-13

**Authors:** Heather Massey, Hannah Denton, Amy Burlingham, Mara Violato, Anna-Marie Bibby-Jones, Rebecca Cunningham, Sandy Ciccognani, Sam Robertson, Clara Strauss

**Affiliations:** 1 Extreme Environments Laboratory, School of Sport, Health and Exercise Sciences, University of Portsmouth, Spinnaker Building, Portsmouth, PO1 2ER UK; 2 R&D Department, Sussex Education Centre, Sussex Partnership NHS Foundation Trust, Nevill Avenue, Brighton & Hove, BN3 7HZ UK; 3 Coventry and Warwickshire Partnership NHS Trust, Coventry, UK; 4 Birmingham and Solihull Mental Health NHS Foundation Trust, Birmingham, UK; 5 Nuffield Department of Population Health, Health Economics Research Centre, University of Oxford, Oxford, UK; 6 School of Psychology, University of Sussex, Pevensey Building, Brighton & Hove, Falmer BN1 9QH UK

**Keywords:** Open water swimming, Sea, Lake, River swimming, Cold water swimming, Cold water, Wellbeing, Depression, Anxiety, Health-related quality of life, Health and social care resource use, Social prescribing, Nature-based interventions, RCT

## Abstract

**Background:**

Depression is common and the prevalence increasing worldwide; at least 1 in 10 people will experience depression in their lifetime. It is associated with economic costs at the individual, healthcare and societal level. Recommended treatments include medication and psychological therapies. However, given the long waiting times, and sometimes poor concordance and engagement with these treatments, a greater range of approaches are needed. Evidence for the potential of outdoor swimming as an intervention to support recovery from depression is emerging, but randomised controlled trials (RCTs) evaluating clinical and cost-effectiveness are lacking. This study seeks to investigate the feasibility of conducting a definitive superiority RCT, comparing an 8-session outdoor swimming course offered in addition to usual care compared to usual care only, in adults who are experiencing mild to moderate symptoms of depression. Feasibility questions will examine recruitment and retention rates, acceptability of randomisation and measures, and identify the primary outcome measure that will inform the sample size calculation for a definitive full-scale RCT. This study will also explore potential facilitators and barriers of participation through evaluation questionnaires, focus-group discussions and interviews.

**Methods/design:**

To address these aims and objectives, a feasibility superiority RCT with 1:1 allocation will be undertaken. We will recruit 88 participants with mild to moderate symptoms of depression through social prescribing organisations and social media in three sites in England. Participants will be randomised to either (1) intervention (8-session outdoor swimming course) plus usual care or (2) usual care only. Both groups will be followed up for a further 8 weeks.

**Discussion:**

If findings from this feasibility RCT are favourable, a fully powered RCT will be conducted to investigate the clinical- and cost-effectiveness of the intervention. Findings from the definitive trial will provide evidence about outdoor swimming for depression for policymakers and has the potential to lead to greater choice of interventions for adults experiencing symptoms of depression.

**Trial registration:**

Current controlled trial registration number is ISRCTN 90851983 registered on 19 May 2022.

**Supplementary Information:**

The online version contains supplementary material available at 10.1186/s40814-023-01358-3.

## Background

Globally, depression has a lifetime prevalence of 10.8% [1], with the rate significantly increasing during the Covid-19 pandemic [2]. The total annual cost of mental ill health in the UK is at least £117.9 billion [3]. This continues to grow nationally and globally [4].

Treatment guidelines recommend a range of psychological therapies, medication and physical activity as evidence-based interventions for depression [5, 6]. However, outcomes from these interventions are variable and do not appear to be reducing the prevalence [7]. Psychological therapy and medications have modest recovery rates (45–50%) [8] with both low adherence to medication [9] and talking therapy completion rates [10]. Exercise as a treatment for depression is also recommended and appears to be effective for some [11]. However, only 66% of males and 58% of females meet UK government recommended levels of physical activity (150 min of moderate activity or 75 min of vigorous activity per week) [12]. Therefore, a greater range of approaches are required in addition to medication, talking therapies or the more standard forms of exercise.

There is growing evidence that access to the natural environment (green and blue spaces) is linked to improving mental health [13, 14]. Green exercise initiatives show great promise in improving wellbeing. Mind commissioned an evaluation of 25 ecotherapy projects, in which participants were encouraged to be active outdoors and found measurable improvements with 7 out of 10 people experiencing significant increases in wellbeing, social inclusion, healthier lifestyles, greater connection with the natural world and adoption of environmentally informed behaviours [15]. However, not all types of green and blue exercise will equally engage all people, for instance, due to injury or poor motivation to do the activity. Therefore, a range of nature-based activities are required so that people can find and choose a preferred activity with which they can best engage.

There is growing evidence that access to green and blue spaces is linked to improving mental health [13, 14]. Green exercise initiatives have shown promise in improving wellbeing. MIND [15] commissioned an evaluation into over 25 ecotherapy projects, in which participants were encouraged to be active outdoors and found measurable improvements with 7 out of 10 people experiencing significant increases in wellbeing, increased social inclusion, healthier lifestyles, greater connection with the natural world and adoption of environmentally informed behaviours. However, not all exercises will engage all people, for instance, due to injury or poor motivation to do specific activities (reported by members of the patient and public inclusion group for the current study). Therefore, a range of activities are required, including those which are nature based, so that people can find an activity with which they can engage. Qualitative research has also indicated that undertaking nature-based activity within water offers additional benefits that could contribute to improved mental health and wellbeing. These include being “present” [16], a sense of escape from everyday life [17] and developing resilience [18] as a risky activity becomes an enabling one [19]. The experience of activity in water has been described as “transformative, connecting and reorientating” as participants enjoy feeling better after a swim, a sense of connection to nature and others and developing different perspectives about themselves and the world [20]. There are a range of possible physiological mechanisms as a result of activity in water that may contribute to an impact on mental health (e.g. cold water vagus nerve stimulation [21], “post swim” stress hormone responses [22], cross-adaptation and reduced cortisol responses [23, 24] and reductions in inflammatory responses [25]). As outdoor swimming is a nature-based activity that is increasing in popularity [26], and has the potential to affect a range of outcomes including mental health [27–29], it is worthy of further study.

The water around the UK is cold all year, and therefore, open water swimming in the UK is considered cold water swimming. Typically, summer water temperatures are between 15 and 20 °C, and conversely, water temperatures can become very cold in winter between 0 and 7 °C [31]. As such, there is a seasonality to outdoor swimming, particularly for those being introduced to the activity, as it is recommended that they start in the summer months [32] when the water temperatures are at their highest. They can then choose to continue as the water cools. As participation in groups is strongly encouraged, for safety reasons, many groups will modify the activity reducing the time in the water during colder months; wear neoprene gloves, boots and a wetsuit in winter [32], and try different locations where the water temperature may be higher, and some may also decide not to swim but maintain contact with the group during this time. These are all potential mitigations to the seasonal nature of the activity. As the effect on depressive symptoms of such an intervention has not been investigated by a robust RCT, the potential mechanisms of the effect have also not been fully investigated; whilst outdoor swimming may be seasonal in the UK, it does not mean all outdoor swimming activity would stop.

Further trials using robust designs, including RCTs incorporating mixed methods, are required to understand if outdoor swimming has potential as a treatment for depression [33]. As outdoor swimming can also be hazardous [25], well-planned empirical research is needed to determine if it is safe and as helpful as early research suggests, before recommending it as an evidence-based treatment for depression. Likewise, it is essential to determine whether sufficient numbers of people with depression will be interested in participating in the intervention, who it most benefits and if it is less attractive to some members of the population and whether measures can be put in place to address this.

This is a protocol for a feasibility RCT, comparing an 8-session outdoor swimming course offered in addition to usual care, with usual care only, in adults who have mild to moderate symptoms of depression. Our aim is to assess the feasibility of undertaking a definitive superiority RCT, comparing an 8-session outdoor swimming course offered in addition to usual care compared to usual care only, in adults who have mild to moderate depression. Objectives are as follows: (1) to determine recruitment and retention rates, acceptability of randomisation and measures, and identify the primary outcome measure that will inform the sample size calculation for a definitive full-scale RCT and (2) to understand potential facilitators and barriers of participation through evaluation questionnaires, focus-group discussions and interviews.

## Methods/design

### Design and sample size

**Fig. 1:**
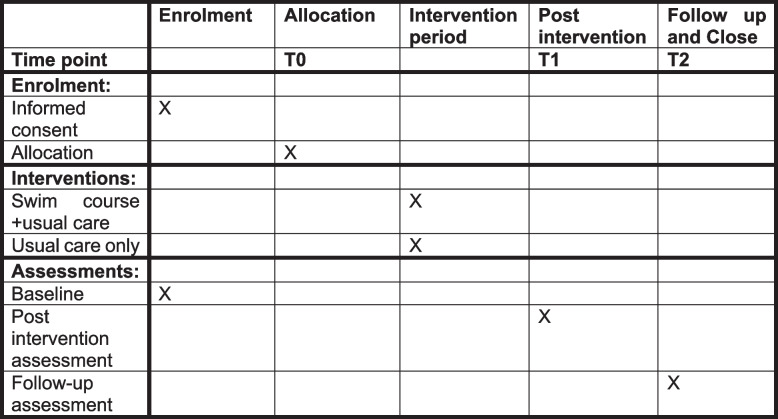
Schedule of enrolment, interventions and assessments

This is a parallel-group, pragmatic superiority feasibility RCT with 1:1 allocation to an 8-session outdoor swimming course plus usual care or usual care only. Using random permuted blocks, we will randomly allocate 88 people meeting eligibility criteria to receive the eight 1-h sessions of a swim course provided by trained and experienced outdoor swim coaches, on top of usual care or usual care only. Stratification is by location (three sites in England). Participants will complete measures at baseline (T0), post swim course or the equivalent time point for the usual care only group (T1) and again after a further 8 weeks (T2) for follow-up (see Fig. 1).

The sample size was based up on Teare et al.’s recommendation for pilot studies [34], where a total of 35 participants would be required per arm. To account for 20% drop-out, a total of 88 participants will be required. This drop-out rate is an estimate based on our previous single-arm study [28].

Evaluation questionnaires, semi-structured focus groups and interviews will be conducted with participants from both arms (at T2) to gain further understanding of barriers and facilitators to recruitment and retention in the study and, for those in the swimming arm, the barriers and facilitators to the swimming courses. Anyone who chooses not to participate will also be invited to complete a questionnaire and, if they had been consented to the study, attend an interview. In addition, social prescribers referring potential participants and the swim coaches providing the courses will be invited to attend focus groups to share their perspectives.

The study design was informed in consultation with a public and patient involvement (PPI) group and supported by the PPI co-applicant (SC). Members of the PPI group had previously participated in introductory swim courses, set up introductory swim courses for people with mental health difficulties or had lived experience of depression. The PPI group were instrumental in guiding the study team to focus on the participants’ journey through the trial, developing the participant information sheets and helping to inform the recruitment strategy.

### Participants

Participants will be recruited through social prescribing teams local to the swim locations and through self-referral to the study following advertising on social media. Social prescribing link workers, usually employed or commissioned through general practice in the National Health Service (NHS) in England, dedicate time to supporting people with their mental health needs and promoting awareness of activities that their patients can participate in to help themselves. NHS England’s mental health long-term plan [35] recommended the introduction of 1000 new social prescribers by the end of 2020/2021 and with more to follow.

Inclusion criteria are that participants will be as follows:

1. Aged 18 years or older
2. Able to give informed consent to participate
3. Have mild to moderate severity depression, as determined by the 9-question Patient Health Questionnaire ([PHQ-9] scores 5–19 inclusive)
4. Have a self-reported ability to swim a minimum distance in a heated pool. This varies by swim location, which differs between the 3 sites: (1) sea (50 m, 2 length of a normal swimming pool); (2) lake (25 m, 1 length of a normal swimming pool); and (3) no swimming experience is required for outdoor unheated swimming pool (lido).

People will be excluded on the following basis:

1. Risk of suicide or recent suicidal intent or attempts or self-harm requiring medical treatment
2. Other mental health problems to a severe degree
3. Not able to speak English to a level that would enable them to understand safety instructions
4. History of cardiac abnormalities (e.g. ischaemic heart disease/angina and congestive heart failure)
5. Immediate (first degree) relative history of cardiac events (GP consultation will be required for inclusion).
6. Respiratory conditions triggered by cold such as poorly controlled exercise-induced asthma
7. Coldwater urticaria (a skin reaction to cold that appears within minutes after cold exposure)
8. Non-freezing cold injuries/Raynaud’s

### Measures

#### Demographics

A questionnaire will be completed by participants to collect key demographics (e.g. age, gender).

#### Primary feasibility measures

- Participant recruitment rates (numbers recruited per week)
- Study retention (percentage of participants with 95% confidence intervals)
- Intervention retention (percentage of intervention participants attending at least 4 of the 8 swim sessions with 95% confidence intervals)
- Data completeness (%)

#### Secondary feasibility measures

- Experience of participation for participants, link workers and coaches
- The number and type of serious adverse reactions

#### Clinical measures

The following measures will be administered at T0, T1 and T2:

- Depressive symptom severity*.* The PHQ-9 will be completed during the consent appointment to determine depressive symptom severity. The PHQ-9 is widely used in primary health care research [36]. It is a 9-item self-report measure of depression severity used with good sensitivity and specificity. Items are rated on a 4-point scale. Scores 5–9 are considered mild, 10–14 moderate, 15–19 moderately severe and 20+ severe [37].
- Generalised anxiety will be measured with the Generalised Anxiety Disorder-7 (GAD-7) [38], a 7-item measure of generalised anxiety. Items are rated on a 4-point scale, and the measure has excellent psychometric properties [39].
- Wellbeing will be measured with the short version of the Warwick Edinburgh Mental Well-being Scale [40], which consists of seven questions rated on a 5-point scale designed to measure wellbeing. The scale has good psychometric properties and is used widely [41, 42].

#### Potential mechanisms

The following measures will be administered at T0, T1 and T2:

- Connectedness to nature will be measured using the connectedness to nature scale [43], which consists of 15 questions rated on a 5-point scale validated scale for measuring connectedness to nature. This will be administered at time 0, time 1 and time 2.
- Loneliness will be measured using three questions rated on a 3-point scale and one question on a 5-point scale, and the three-question survey has good psychometric properties [44] and is used widely in conjunction with the final question [45].
- Self-compassion will be measured using the 20 question Sussex-Oxford Compassion for Self-Scale (SOCS-S) that is rated on a 5-point scale [46] The scale has excellent psychometric properties.
- Mindfulness will be measured using the 15-item version of the Five-Facet Mindfulness Questionnaire [47]. This has excellent psychometric properties.

The PPI group was consulted on the use of these measures and the most appropriate formats. As a result, surveys will be offered as an online form available via e-mailed links, surveys completed by phone with the participant or in paper version, whichever the participant finds most useful and acceptable.

#### Health economic measures

The following health economics outcomes and measures will be collected at T0, T1 and T2.

*Health-related quality of life (HRQoL)*: HRQoL will be measured using the following:

- The EQ-5D-5L [48] — This is a well-validated, generic, preference-based measure of HRQoL in adult populations, widely used across disease areas, including mental health. It includes five dimensions covering domains of everyday life, i.e. mobility, self-care, usual activities, pain/discomfort and anxiety/depression, each with five-ordered levels of response. It is designed to estimate quality-adjusted life years (QALYs) and recommended by the UK National Institute for Health and Care Excellence (NICE) for use in economic evaluations for health technology assessment [49].
- The ReQoL-10 (Recovering Quality of Life-10) [50] — This is a specific measure of HRQoL for people aged 16 years old or more experiencing a wide range of mental health difficulties, including anxiety and depression. The recently developed valuation of the ReQoL-10, i.e. the ReQoL-Utility Index (UI) [51], can be used for estimating QALYs. It will be used in this feasibility study as a sensitivity measure.

*Resource use*: A societal perspective for resource use data collection will be adopted as mental health consequences/costs go beyond the health and social care sectors, e.g. including the labour market. The following questionnaires will be tailored to the target population with input from the PPI group:

- Client Service Receipt Inventory (CSRI) [52] form: A modified version of the CSRI will be used to collect information on participants’ type and intensity of usual care for depression for the duration of the study, as applicable, use of other health and social care resources (e.g. GP visits, medications) and time/cost of travelling to/from treatment locations. This has good agreement with case records [53]. Participants will also be provided with “ad hoc” designed diaries to help recalling of resources used between assessment points.
- Institute of Medical Technology Assessment (i-MTA) Productivity Cost Questionnaire (iPCQ) [54]: An adapted version of the iPCQ will be used to capture the impact of mental health on employment in a paid job and on unpaid work (e.g. household chores).
- “Ad hoc” form/logs: These bespoke form/logs will capture the relevant setup and on-going resources necessary to offer the outdoor swimming courses (e.g. insurance; lifesaving equipment). They will include specific logs for swimming coaches and their supervisors (for recording time spent on training and preparation/delivery of the outdoor swimming course) and logs for link workers (to record time spent advising participants).

#### Intervention evaluation measures and tools

A qualitative component of the study will explore the available evidence to help identify facilitators and barriers to participation in both arms of the trial. The following tools have been developed to support qualitative data collection:

- A questionnaire for people who choose not to participate
- A semi-structured focus group discussion schedule for social prescribers
- A questionnaire and semi-structured interview schedule for participants who do not complete the study
- A semi-structured focus-group discussion schedule for swim coaches
- An evaluation questionnaire, semi-structured focus-group discussion and semi-structured interview schedule for participants in the intervention arm
- An evaluation questionnaire, semi-structured focus group discussion and semi-structured interview schedule for participants in the control arm

### Procedure

See Fig. 1 for the SPIRIT [55] schedule of enrolment, interventions and assessments. Participants with mild to moderate symptoms of depression will be sought through social prescribing services and self-referral. The study will be advertised by social prescribers and elsewhere through social media. Informed consent will be obtained from each participant by the recruitment team (HM, AB, RC and AJ). Potential participants will be given a copy of the study participant information sheet and will have the opportunity to discuss the study with the research assistant (RA) by phone or video call before signing the consent form (a copy of which can be obtained from the corresponding author). Written and verbal consent will be received at least 24 h after participants are given the participant information sheet.

To assess the risk at entry to the trial, initial information will be gathered from self-reported online health history questionnaires about the participant’s medical history. The research assistant will also perform the PHQ-9 at the consenting phone call. The health history questionnaire and PHQ-9 will be assessed by the medical team RC and AB (both psychiatrists) who will evaluate the risk. Following this, those deemed at high risk will be contacted by the medical team via a phone call, and those deemed as low risk or where further information is required will be contacted by the research assistant or other members of the research team either by phone or email and answers discussed with the medical team if necessary. During the study, if a concern is raised by the participant, swim coaches or a study team member the medical team will contact those involved and recommend the actions to be taken, if any.

Once the participant has consented to participate in the trial, the participant will complete the full set of baseline measures. Measures will be completed online, on paper or with an RA present by telephone depending on participant preference. Participants who do not meet eligibility criteria at the baseline assessment will be referred to social prescribers to be offered alternative activities.

At the end of the baseline assessment, eligible participants will be randomised to either the swim and usual care or usual care only arm. Eligible participants will be randomly allocated using the Sealed Envelope online service [56]. The study statistician will use Sealed Envelope to set up and test the randomisation procedure, incorporating stratification by site using random permuted blocks and 1:1 allocation. The statistician will not have any further involvement in the randomisation process. One of the Co-CI’s (H. M.) will randomise participants by completing the online form with participant’s details. This will immediately show whether the participant is assigned to the swim course intervention plus usual care or usual care only arm. Participants assigned to the intervention will be given details of the swim courses and, where possible, support from social prescribers to attend the sessions. Participants will continue with their usual care and complete the surveys at T0, T1 and T2, with the intervention group participating in 8 outdoor swimming sessions. The quantitative data analysis team (AMBJ and MV) will remain blinded until the quantitative data analysis is complete. The RA-supporting participants to complete measures will remain blinded until all quantitative data is collected.

The outdoor swim courses are led by experienced qualified outdoor swimming coaches who have mental health awareness training. Swim coaches will contact participants before the first session to ensure they know what to bring with them and answer any questions. The swim coaches will contact participants 24 h before each session to support their attendance and provide any additional information e.g. a change of plan due to inclement weather. If possible, social prescribers will support participants to their first session. This mirrors the usual way in which social prescribers provide support for clients attending new activities.

Participants will complete further measures online (with a postal option) at the post intervention time point (T1), or equivalent time point if they are in the usual care group and at 8-week follow up (T2). Participants will have the option to choose whether to complete the T1 and T2 measures with an RA present by telephone (who will be blind to group allocation) or on their own. In the event that an RA is required to be present during T1 and T2 assessments and becomes unblinded, the assessment will be completed by another blinded team member. However, participants will be asked not to reveal which arm of the study they were randomised to. Where T1 and T2 assessments are not completed, participants will be contacted at weekly intervals for up to 1 month to remind them to complete these assessments, unless participants have discontinued from the study.

The qualitative data will be collected using questionnaires, focus groups and interviews. A questionnaire will be sent to those declining to participate and a questionnaire and an invitation to participate in an interview to those who do not remain in the study. The questions are designed to understand any barriers they encountered and to determine any changes that could be made to improve recruitment and retention in the definitive trial. Following completion of recruitment and the swim courses, the social prescribers and swim coaches will all be invited to participate in focus groups to explore their understanding of any facilitators and barriers. Finally, at the end of the follow-up period (T2), all study participants will be sent an evaluation form. This will provide an opportunity to feedback about their experiences of the study; the intervention group evaluation form will also ask about their experience of being on the swim course. They will also all be invited to attend a focus group, to gather further detailed information about their experiences. Six focus-group sessions will be offered: one for participants in the intervention arm and one for controls in each location. Additionally, participants will be invited for an interview if further in-depth exploration of their experience would be useful. Information gathering will be purposive, and analysis will be undertaken alongside data collection and inform when sufficient data has been achieved. Questions were added to the evaluation form and semi-structured interview schedule by the trial lived experience and PPI team to improve the understanding of the experience of the study from a participant perspective.

#### Intervention protocol

Participants randomised to the intervention will be contacted by the swimming coaches leading the 8-session outdoor swimming course prior to the start and given further information and answer any questions. The outdoor swimming courses will be held in three sites in England. The course will run for 8 × 1 h sessions. During the course, participants will also continue with their usual care (medications and/or therapies). The groups will consist of only participants in the research study; the groups will not be open to the general public.

#### Course structure

The focus of these courses will be the safe enjoyment of the water rather than becoming an expert swimmer. The swim coach will phone or text participants the night before each session and meet them outside the facilities. They will walk into the facility together and will be shown where to change and what to bring with them to the water’s edge.

All swimmers will be supervised by the course staff (who have basic life support, lifeguard qualifications and have attended courses on mental health first aid and mental health awareness training), and they will only swim if it is safe to do so. It is not possible to offer a completely standardised course as local conditions will dictate what can be achieved in the sessions.

Outdoor swimming courses should be offered when the water temperatures are at their highest. Therefore, the intervention group will start their swimming course no earlier than the beginning of August and courses will finish no later than the middle of November. The sessions will include basic swimming skills (Lido only), safety, hazard awareness and risk assessment and development of competency to deal with moderate challenges when in the water, including floating and in-water-based skills. However, alternative arrangements and session plans will be available for adverse weather conditions that do not allow the swim to take place at the normal time.

Wetsuits will not be provided as the water will be between 15–20 °C), but participants can use their own wetsuit if they wish. It will be made clear to participants that they should leave the water immediately should they feel uncomfortably cold. Although the session is an hour in length, the maximum in-water time will be 30 min. The remaining 30 min will be split between the pre-swim briefing and post-swim rewarming. After exiting the water, participants will return to the changing areas and change into warm dry clothes. Participants will be encouraged to have a hot drink. Risk assessments will be carried out on the day by the swim coach and lifeguard. Participants will consent for the research team to provide the swim coaches with details of medical conditions, swim experience and emergency contact details. At all times, clients will be monitored closely for signs of hypothermia.

During the course, participants will be supported to find existing groups or form their own so that they can continue to swim safely after completion of the course.

#### Usual care protocol

The usual care only arm will act as the control. They will be treated in accordance with the current NHS care by their medical providers. During the study, they may be taking medications, be on waiting lists for engaging in talking therapies, or participating in other social prescribing activities. Their participation in other interventions will be variable and we will ask them to record any treatments or interventions they engage in. Participants randomised to this group will be given the opportunity to participate in an introductory course of outdoor swimming upon completion of the study to thank them for their participation. This was advised by PPI representatives as a way to increase the likelihood of participants agreeing to the randomisation process and reduce the number who may drop out. Funding for these courses has been secured.

### Intervention adherence and acceptability

Intervention adherence and acceptability will be assessed on the basis of attendance of participants at the swim sessions and rates of survey completions. Further information about the acceptability of the intervention and study design for participants will be gathered through the following: the evaluation forms and interviews with any participants who did not complete the intervention, from evaluation forms, focus groups and interviews with participants who completed the intervention or were in the control group, and from a focus group with the swim coaches.

### Serious adverse event monitoring

A protocol for identifying and independently assessing serious adverse events will ensure that such events are addressed in a timely fashion and responded to, including if a serious adverse event is classified as potentially study related. Serious adverse events and their classification will be reported to the Trial Steering Committee (TSC), and action will be taken as deemed necessary. Where deemed in the best interests of participants, the study intervention may be discontinued.

### Planned data analysis

Participant flow will be presented using a CONSORT diagram [57, 58]. Summary statistics (means, standard deviations, median, interquartile range, counts or percentages) will be provided for all demographics (age, gender, ethnicity, sexual orientation, disability status, religion or belief, relationship status, employment status and level of swimming ability), quantitative outcomes and feasibility measures.

Outcome, potential mechanism and health economic measures will be summarised for each trial arm at each time point. Effect sizes for between-group mean differences for each individual outcome and potential mediator measure will be estimated using linear mixed models post intervention (T1) and at 8-week follow-up (T2). The models will include fixed effects for treatment group (outdoor swimming + usual care, usual care), time (T1, T2) and the group × time interaction, the stratification variable (site) and the measure’s baseline score will be entered as covariates to adjust for potential bias and improve efficiency [59] and random effects will be added to account for repeated measurements on individuals. Parameter estimates with 95% confidence intervals will be presented, and there will be no hypothesis testing.

For PHQ-9 scores, 95% confidence intervals for the between-group difference will be reviewed with respect to the minimal clinically important difference (MCID) based on Kounali et al.’s [60] threshold of 1.7 for the difference between feeling better and feeling the same. We will proceed to a full trial if the 95% confidence interval for the between-group difference includes an effect in favour of the intervention arm of 1.7 PHQ-9 points at post-intervention, if the other feasibility parameters are met.

With regard to the economic outcomes, for the two economic measures of HRQoL, i.e. the EQ-5D-5L [48] and the ReQoL-10 [50], utility scores will be calculated using validated algorithms [49, 51, 61, 62] for the treatment and the control groups to explore which measure is more sensitive to changes over time. The use of all resources/services will be reported by trial arm, stratified by use/service type, as the mean, standard deviation, range and the percentage of the sample for each arm that had at least one contact/use, as applicable. Statistical analyses will be conducted using Stata version 16 (StataCorp), whilst Stata version 17 will be used for the health economics analyses.

Suitability and acceptability of the outcome, potential mechanism and health economic measures for a fully powered trial will be assessed based on measurement completion rates and participant’s feedback gained from evaluation forms, focus groups and interviews. Study findings will inform the selection of the primary outcome and inform the sample size of a future full-scale RCT should this study be deemed feasible.

Qualitative data will be analysed to identify facilitators and barriers to engagement with the study and with the intervention which will highlight areas that could be changed to improve acceptability in a full trial.

#### Data entry accuracy and missing data

We aim to minimise missing data and data entry inaccuracies at the point of collection. The Qualtrics online survey software used to collect all data will automatically flag any unanswered questions, giving participants the chance to answer these. If a participant would prefer not to answer a question, they can leave it unanswered for a second time, and the survey will proceed to the next page. At the point of analysis, data will be summarised to look at patterns of missingness, but it will not be replaced. Anonymised data will be stored on the Qualtrics platform and on password-protected NHS and university computers. Personal data will be stored securely on password-protected NHS computers and servers.

#### Planned interim analysis and stopping rules

No interim analysis has been planned. The trial will be paused or stopped if deemed necessary by the TSC.

#### Multiple testing

As this feasibility study is exploratory and there is no hypothesis testing, there will be no adjustment made to the analysis for multiple testing.

#### Qualitative data analysis

Qualitative data will be analysed using thematic analysis [63], informed by a critical realist perspective, with the aim of better understanding facilitators and barriers of participation for each research arm. Multiple perspectives will be included as insights will be gathered from social prescribers, swim coaches, potential participants declining the study, participants leaving the study and all participants completing the study. Focus-group discussions and interviews will be transcribed and included in the data set along with free text answers in evaluation forms. The analysis will be inductive, so the questions asked will not inform the themes and undertaken by one of the researchers (HD) and the RA using NVivo. The rest of the research team and the PPI will act in the role of critical friends to encourage reflexivity.

### Criteria for success

This feasibility trial, if successful, will inform the development of a full-scale RCT. Criteria for success of the feasibility work will include the following:

• 80% trial retention at T1 for the PHQ-9
• 80% attending four or more swim sessions
• 80% data completeness
• Collection of data on the experience of participation from at least 20% of participants
• Findings indicate that taking part in this study was a positive experience for participants, link workers and coaches.

## Discussion

The popularity of open water swimming is growing [30], and studies report benefits to mental health and wellbeing [20, 27–29]. At present, the evidence is limited to pilot studies, qualitative research and surveys, and therefore, the justification for scaling up the use of this activity as an alternative to other forms of treatment for depression is required.

This feasibility RCT, to determine the impact of an outdoor swimming intervention, will be undertaken with the aim of informing a definitive full-scale RCT. There is not enough known about the potential clinical- and cost-effectiveness of outdoor swimming as an intervention for people living with depression. Our study aims to assess the feasibility of undertaking a definitive full-scale superiority RCT, comparing an 8-session outdoor swimming course offered in addition to usual care, compared to usual care only, in adults who have mild to moderate symptoms of depression. Objectives are as follows: (1) to determine recruitment and retention rates, acceptability of randomisation and measures, and identify the primary outcome measure that will inform the sample size calculation for a definitive full-scale RCT, and (2) to understand potential facilitators and barriers of participation through evaluation questionnaires, focus-group discussions and interviews.

Findings from the qualitative analysis will highlight facilitators and barriers to engagement in both the intervention itself and the study protocol. This will lead to recommendations for maximising engagement in a full-scale definitive RCT.

The results of the feasibility RCT will be used to develop a protocol for a definitive full-scale RCT, which will be undertaken providing feasibility aims are met, or if not met could be addressed. The results of the full-scale definitive RCT will provide evidence regarding the clinical and cost effectiveness of open water swimming for depression. In addition, this potential future trial would explore the mechanisms involved in terms of “what provision works, and for whom?” as discussed by Garside [33]. As well as supporting the expansion of the range of nature-based socially prescribed activities, this would ensure that such interventions are targeting mechanisms of action as specifically as possible. Ultimately, we hope that this feasibility study will pave the way to develop the evidence base for open water swimming for depression, so that people living with depression may potentially have a greater range of options to support their recovery.

### Trial status

At the time of manuscript submission, recruitment for this study was ongoing. Recruitment started on 6th July 2022, is not yet complete and is due to be completed on 6th September 2022. This is protocol version 1.7 (02.07.22). The trial sponsor is the Sussex Partnership NHS Foundation Trust (ResearchGovernance@sussexpartnership.nhs.uk).

## Supplementary Information

**Additional file 1.**

## Data Availability

The datasets created for the current study will be available from the corresponding author on reasonable request.

## Abbreviations

CSRI: Clinical Service Receipt Inventory
DEFRA: Department of Environment, Food and Rural Affairs
DSMB: Data Safety Monitoring Board
EQ-5D-5L: EuroQol-5-dimension-5-level instrument
GAD-7: Generalised Anxiety Disorder 7
IAPT: Improving access to psychological therapies
iPCQ: i-Productivity Cost Questionnaire
NICE: National Institute of Health and Care Excellence
PHQ-9: Patient Health Questionnaire-9
PPI: Public and patient involvement
RA: Research assistant
RCT: Randomised control trial
ReQoL-10: Recovering Quality of Life-10 instrument
SOCS-S: Sussex-Oxford Compassion for Self-Scale
sWEMWBS: Short version of the Warwick Edinburgh Mental Well-being Scale
TSC: Trial Steering Committee
